# Coefficient-metric gradient-based digital wavefront correction for full-field swept-source optical coherence tomography

**DOI:** 10.1364/BOE.592421

**Published:** 2026-03-24

**Authors:** Guozheng Xu, Jem Love, Thomas J. Smart, Destiny Hsu, Myeong Jin Ju, Marinko V. Sarunic

**Affiliations:** 1 Department of Medical Physics and Biomedical Engineering, University College London, London WC1E 6BT, United Kingdom; 2 Institute of Ophthalmology, University College London, London WC1E 6BT, United Kingdom; 3 School of Engineering Science, Simon Fraser University, Burnaby, BC V5A 1S6, Canada; 4 School of Biomedical Engineering, University of British Columbia, Vancouver, British Columbia V6 T 1Z3, Canada; 5 Department of Ophthalmology & Visual Sciences, University of British Columbia, Vancouver, British Columbia V5Z 3N9, Canada

## Abstract

Full-field swept source optical coherence tomography (FF-SS-OCT) achieves fast acquisition of depth-resolved volumetric data. It preserves the phase stability across *en face* features, which enables digital wavefront correction during post-processing. Traditional digital wavefront correction methods, such as coordinate search, are reliable but time-consuming. We present CoMGrad, a novel GPU-accelerated coefficient-metric gradient-based optimisation method for digital wavefront correction for FF-SS-OCT. CoMGrad utilises the differentiability of the mapping from the Zernike coefficients to the image quality metric. Reverse-mode automatic differentiation and the Adam optimiser were used to increase time efficiency. Theoretical analysis and *in vivo* aberration correction tests on human retinas demonstrate that CoMGrad provides more than a tenfold increase in time efficiency compared to coordinate search.

## 1. Introduction

Optical coherence tomography is a powerful technology that utilises low-coherence interferometry to produce non-invasive cross-sectional imaging of biological samples [1]. Ocular imaging is a common application for OCT, as it provides non-invasive and depth-resolved imaging of the retina [2]. Fourier-domain OCT resolves depth information through spectral analysis of the interference pattern, which provides sensitivity and speed advantages over time-domain OCT [3–9].

Recently, full-field swept-source OCT (FF-SS-OCT) has been developing rapidly. Compared to point-scanning swept-source OCT, which uses a one-dimensional photodetector to detect each point being scanned, FF-SS-OCT drastically increases the speed of three-dimensional volumetric data acquisition by imaging a two-dimensional cross-sectional object layer onto an area camera [10–12]. This higher speed introduces several advantages, such as clinically-friendly operation, a larger field of view (FOV), and phase-stable volumes of the retina. A main limitation of FF-SS-OCT is optical crosstalk, which effectively reduces the spatial resolution, especially at greater depths in scattering samples. This is caused by the spatial coherence of the laser source illumination. The development of spatiotemporal optical coherence tomography (STOC-T) overcomes this limitation by introducing both spatial and temporal incoherence through a long optical fibre to prevent crosstalk [13]. STOC-T, combined with post-processing and digital wavefront correction (DWC), allows for axial and lateral resolutions of ∼5 µm over the entire thickness of the retina without any mechanical scanning [14–17].

To achieve higher lateral resolution in retinal imaging, adaptive optics (AO) methods have been incorporated into OCT systems [18–23]. Hardware-based AO measures the wavefront distortion using a Shack-Hartmann or pyramid wavefront sensor and applies the phase conjugate to a deformable mirror (DM) or other wavefront modification devices to cancel out the wavefront distortion [24–27]. Alternatively, some AO methods correct the wavefront distortion by optimising certain image quality metrics instead of directly measuring the wavefront using wavefront sensors, known as wavefront sensor-less adaptive optics (WFS-less AO). WFS-less AO avoids the disadvantages of wavefront sensors, such as an ill-defined reference plane, alignment, system complexity, and non-common path aberrations [28,29]. However, WFS-less AO methods are usually more time-consuming due to the iterative optimisation process, which commonly treats the distorted wavefront as a superposition of modal basis functions and optimises the modal coefficients, including Zernike mode hill climbing (ZMHC), stochastic gradient approximation methods like stochastic parallel gradient decent (SPGD), and model-based methods [30–35]. Artificial intelligence has also been applied to WFS-less AO to accelerate the optimisation process [36–41].

Another category of AO methods utilises the phase-stable data acquired from FF-SS-OCT systems, such as STOC-T, for post-processing digital wavefront correction. Traditional WFS-less AO numerical optimisation methods can be effectively applied to DWC, such as coordinate search, and the difference is that DWC is performed computationally, without live interaction with a DM or the sample [42–45]. Since FF-SS-OCT data preserves the phase information of the complex field, methods such as sub-aperture correlation-based numerical phase correction can be used, provided that the complex object field information can be extracted without the need for knowledge of system parameters [46,47]. Improvements of robustness and automation by using randomised subapertures have also been reported [48,49]. These approaches provide robust alternatives to metric-based optimisation methods.

In this paper, we present a new numerical optimisation DWC method for phase-stable data acquired by the STOC-T system: coefficient-metric gradient-based digital wavefront correction (CoMGrad). CoMGrad utilises the mathematical differentiability of the mapping from Zernike coefficients to image quality metrics. Reverse-mode automatic differentiation is applied to generate gradients for each Zernike coefficient at the cost of only two passes of the coefficient-metric chain, irrespective of the number of modes used [50,51]. With first-order gradient-based optimisation, CoMGrad performs DWC with knowledge of analytical local derivatives of optimisation parameters at low computation load, highlighting its speed advantage over zeroth-order optimisation methods such as ZMHC and SPGD in the sensor-less AO field, where end-to-end gradients of the optimisation coefficients are typically not directly available. The Adam optimiser has been used to update the coefficients to find the optimal wavefront shape based on these gradients [52]. Theoretical analysis and experiments were conducted in comparison to a widely used and reliable DWC method, coordinate search, to demonstrate the speed advantage of CoMGrad. Results show that CoMGrad can achieve more than a tenfold improvement in speed compared to coordinate search while maintaining similar or even superior aberration correction performance.

## 2. Methods

### 2.1. STOC-T system & post-processing pipeline

**Fig. 1.**
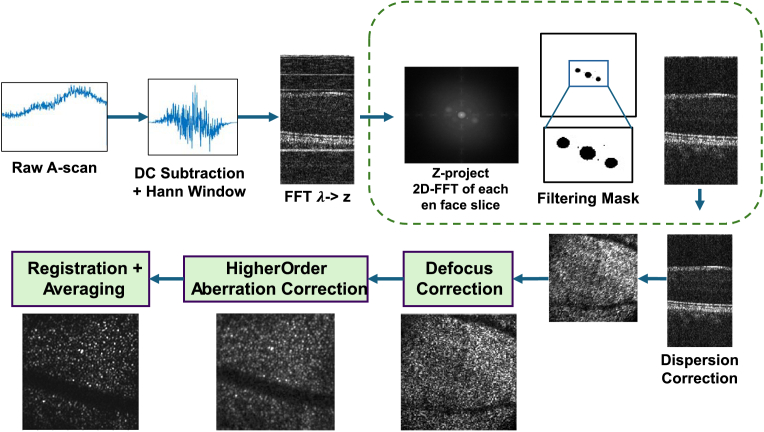
STOC-T post-processing pipeline.

The optical system used in this study, the STOC-T instrument (InCellVu, S.A.), is a replica of the instrument described in [14]. The post-processing pipeline for STOC-T data is illustrated in Fig. 1. Post-processing begins with DC subtraction and the application of a Hanning window to each individual A-scan within a volume. Subsequently, a Fourier transform is performed to convert the spectral domain interference signals into the spatial domain. A two-dimensional filter is then applied to the spatial frequency domain data to suppress large speckles originating from the multimode fibre as well as internal reflections from the STOC-T system [14]. Following filtering and dispersion compensation, the volumetric data is prepared for DWC. The DWC process starts with the removal of defocus of target *en face* slices. The initial correction reduces modal interaction and increases the convergence speed for subsequent higher-order aberration corrections. After correcting higher-order aberrations, volumes are registered and averaged to improve the signal-to-noise ratio (SNR) and overall visualisation. In this work, CoMGrad is specifically implemented during the process of the higher-order aberration correction stage.

### 2.2. Image metric-based DWC

For STOC-T, three-dimensional complex wave field is detected in the image plane. The three-dimensional data acquired by STOC-T are phase-stable in the cross-sectional layers after Fourier transform reconstruction. Assuming the cross-sectional *en face* slice in the image plane is $u_{img}$ , the Fourier transform of $u_{img}$ reveals the spatial frequency domain features, which can be expressed by:

(1) $$\begin{array}{l} F\{u_{img}\}={\tilde{u}}_{obj}\times \exp\{j\phi_{0}\}, \end{array}$$

where ${\tilde{u}}_{obj}$ is the spatial frequency domain spectrum of the spatial domain object without wavefront aberrations, and $\phi_{0}$ represents the wavefront distortion of $u_{img}$ . The physical meaning of the Fourier transform manipulation in Eq. (1) is to transform the complex *en face* field in the image plane into the spatial frequency domain, where the wavefront aberration appears as a multiplicative phase term acting on the object spectrum. This representation enables DWC by compensating for the phase distortion.

DWC applies the phase conjugate $\phi_{c}$ to $F\{u_{img}\}$ to correct the wavefront distortion, which can be modelled as a superposition of Zernike polynomials $Z_{i}$ weighted by their corresponding Zernike coefficients $c_{i}$ :

(2) $$\begin{array}{l} \phi_{c}=\overset{N}{\underset{i=1}{\sum }}c_{i}Z_{i}, \end{array}$$

where *N* is the total number of Zernike modes [53–55].

The aberration-corrected *en face* image intensity $I_{c}$ is the inverse Fourier transform of $F\{u_{img}\}$ multiplied by the phase correction term:

(3) $$\begin{array}{l} I_{c}={\left|F^{-1}\{{\tilde{u}}_{obj}\times \exp\{j\phi_{0}\}\times \exp(-j\phi_{c})\}\right|}^{2}. \end{array}$$

Image quality is evaluated by image sharpness, defined as the sum of squared pixel intensities of $I_{c}$ [56–58]:

(4) $$\begin{array}{l} M=\overset{N_{p}}{\underset{p=1}{\sum }}{\left|I_{c}(p)\right|}^{2}, \end{array}$$

where *p* represents each pixel and $N_{p}$ is the total number of pixels of $I_{c}$ . DWC is then performed on individual *en face* images by optimising this image sharpness metric. During optimisation, the metrics are normalised by the metric of the corresponding *en face* image before DWC. This normalisation provides a consistent metric scale and ensures a straightforward interpretation of image quality improvement during DWC. No additional intensity normalisation across patches, volumes, or datasets is applied. Consequently, metric values obtained from different *en face* images are not intended to be directly comparable.

The sharpness metric used here is widely adopted as an image-quality criterion in aberration correction. Alternative metrics, such as Shannon entropy, have also been applied to FF-SS-OCT DWC [59]. Although the sharpness metric may be sensitive to speckle or localised bright artefacts, in our experiments, it produced optimisation behaviour comparable to that obtained with Shannon entropy. CoMGrad could also benefit from improved image-metric formulations that are more robust to speckle and imaging artefacts, provided that the metric remains differentiable. The basic principle of image metric-based DWC is to maximise the image quality metric *M* by optimising the Zernike coefficients:

(5) $$\begin{array}{l} {\left\{c_{1},c_{2},\ldots ,c_{N}\right\}}_{opt}=\underset{\left\{c_{1},c_{2},\ldots ,c_{N}\right\}}{\underbrace{argmax}}\left\{\overset{N_{p}}{\underset{p=1}{\sum }}{\left|F^{-1}\left\{{\tilde{u}}_{obj}\exp\{j\phi_{0}\}\exp\left(-j\overset{N}{\underset{i=1}{\sum }}c_{i}Z_{i}\right)\right\}\right|}^{4}\right\}. \end{array}$$

### 2.3. CoMGrad pipeline

Since STOC-T data are extremely large due to their three-dimensional nature, post-processing can be computationally demanding. To identify the optimal Zernike coefficients that yield the best image quality, traditional zeroth-order optimisation methods rely on evaluations of image quality metrics, without direct access to the analytical gradient of the coefficient-metric model. Although those methods are robust and stable in terms of optimisation performance, they can become slow and computationally intensive when applied to gigabytes of volumetric data, in stark contrast to the data acquisition process, which is completed within seconds.

We present a new optimisation method, termed CoMGrad, which leverages gradients of the coefficient-metric objective function. Equation (4) can be rewritten as:

(6) $$\begin{array}{l} M(c_{1},c_{2},\ldots ,c_{N})=\overset{N_{p}}{\underset{p=1}{\sum }}{\left|F^{-1}\left\{{\tilde{u}}_{obj}\exp\{j\phi_{0}\}\exp\left(-j\overset{N}{\underset{i=1}{\sum }}c_{i}Z_{i}\right)\right\}\right|}^{4}. \end{array}$$

Notably, all parameters in this objective function are known, and all involved operations, including summation, multiplication, exponentiation, and Fourier transformation, are differentiable. Consequently, the objective function is differentiable with respect to all Zernike coefficients. This differentiability is not directly applicable in hardware-based WFS-less AO systems, where image quality metrics are obtained by applying discrete wavefront perturbations to a deformable mirror and acquiring the resulting images, rather than being derived from a single continuous analytical model. While gradient-like optimisation can still be performed via finite-difference methods or stochastic approximations, end-to-end backpropagation through the physical system is not directly applicable.

**Fig. 2.**
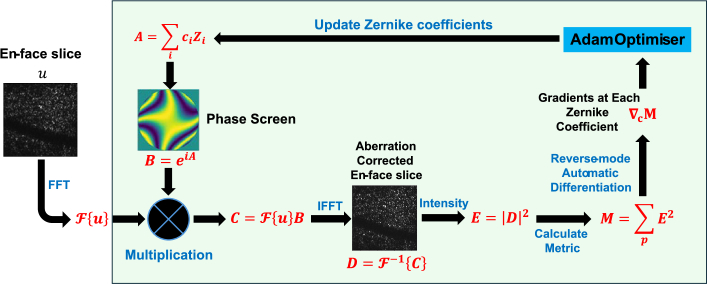
Workflow of CoMGrad for STOC-T. The steps within the green box are iterative. Intermediate states are indicated by red bold equations.

Figure 2 illustrates the workflow of CoMGrad. The intermediate states involved in computing the image sharpness metric from the Zernike coefficients are explicitly shown. The workflow consists of several consecutive operations: modal superposition *A*, phase screen generation *B*, multiplication with the spatial frequency domain image *C*, inverse Fourier transform *D*, intensity generation *E*, and metric calculation *M*. Notably, all these operations are differentiable. The image quality metric is first evaluated in the forward pass using the current Zernike coefficients, during which the intermediate states are stored to evaluate the local partial derivatives of operators. These stored values allow the backward pass to numerically compute the chain rule, projecting the final metric gradient back through the computational graph to the Zernike coefficients. Subsequently, the gradient map $G_{p}$ is obtained via a vector–Jacobian product (VJP) using reverse-mode automatic differentiation [50,51]:

(7) $$\begin{array}{l} G_{p}=\frac{\partial M}{\partial E}\frac{\partial E}{\partial D}\frac{\partial D}{\partial C}\frac{\partial C}{\partial B}\frac{\partial B}{\partial A}=(\nabla_{A}M)(p). \end{array}$$

The VJP propagates sensitivity from the scalar metric *M* backwards through the non-local propagation operators, yielding the pixel-wise phase gradient map $G_{p}$ . $G_{p}=(\nabla_{A}M)(p)$ represents the partial derivatives of the metric *M* with respect to each pixel of the Zernike polynomial superposition *A*.

Since $A(p)=\underset{i}{\sum }c_{i}Z_{i}(p)$ and $\partial A(p)/\partial c_{i}=Z_{i}(p)$ , the gradient with respect to each Zernike coefficient $\nabla_{c_{i}}M$ is then calculated by summing the pixel-wise products between the sensitivity map $G_{p}$ and the corresponding Zernike polynomial:

(8) $$\begin{array}{l} \nabla_{c_{i}}M=\text{Re}\left((\nabla_{A}M)(p)\frac{\partial A(p)}{\partial c_{i}}\right)=\text{Re}\left(\underset{p}{\sum }G_{p}Z_{i}(p)\right), \end{array}$$

where $Z_{i}(p)\;$ denotes the value of the *i*-th Zernike polynomial at pixel *p*. Using the resulting gradient vector, the image quality metric can be optimised with gradient-based optimisers such as Adam, which is widely used in machine learning for updating deep neural network weights.

In this framework, the computational cost of obtaining *N* partial derivatives is dominated by the two Fourier transforms required for the forward-pass metric calculation and the reverse-mode automatic differentiation. Although the projection of the gradient sensitivity map onto the Zernike polynomials scales with the number of phase pixels and modes, it is computationally efficient because it consists of a single real-valued matrix–vector multiplication that can be executed very efficiently on modern CPUs and GPUs, in contrast to the FFT-based forward and adjoint propagations. As a result, the overall computational cost is largely insensitive to the number of Zernike modes, making CoMGrad efficient even when a large number of modes is required to correct higher-order aberrations. The implementation of CoMGrad uses the built-in automatic differentiation framework of PyTorch. Complex-valued operations and gradients in the computation chain are handled internally in PyTorch, while the optimisation variables (the Zernike coefficients) remain real-valued.

### 2.4. Optimisation setup & iteration scheme

To demonstrate the performance of CoMGrad, we chose the coordinate search algorithm for comparison due to its stable performance in AO optimisation. In this section, the optimisation setup and iteration schemes for CoMGrad and coordinate search are discussed.

First of all, a radially decreasing correction limit was applied to all Zernike coefficients for both methods. The second radial order was assigned an initial coefficient limit of [−0.5,0.5] µm, and the limits for higher orders decreased exponentially with the radial order, with a base of 2.5. The minimum limit was [−0.005,0.005] µm. These constraints were imposed experimentally to restrict the correction to a physiologically realistic range of ocular aberrations and to prevent excessive phase modulation from higher-order modes, which can otherwise lead to non-physical solutions such as artificial energy concentration into isolated bright spots. The estimated wavefront distortion from a human retinal imaging session is presented in the experimental results, supporting that these coefficient limits are sufficient for correcting ocular aberrations and potential system aberrations. Nevertheless, the coefficient limits are programmable parameters that can be tuned for improved correction performance.

For coordinate search, multiple search points were used for each mode to determine the optimal Zernike coefficient for that mode. The coefficient corresponding to the maximum metric was recorded until all Zernike modes had been searched. The search across all modes was considered one round. After each round, the estimated Zernike coefficients were updated to the coefficients with the maximum metrics obtained in that round. The optimisation in the following round started from the correction result of the previous round. Multiple rounds were implemented to improve correction accuracy and to mitigate modal interactions.

For the search of each mode, seven search points were used. The search range of coefficients for each mode was determined by two factors: the mode’s radial order and the current round of the search. Within each round, the search range for each mode decreased exponentially with radial order, with a base of 1.3. Across rounds, the search ranges for all modes were multiplied by another exponentially decreasing factor with a base of 1.3. The search range for the 2^nd^ radial order at the first round was [−0.2,0.2] µm. The search range $R_{k}^{r}$ of a Zernike mode with radial order *k* at round *r* was defined as:

(9) $$\begin{array}{l} R_{k}^{r}=[-0.2\times 1.3^{-(r+k-2)},0.2\times 1.3^{-(r+k-2)}]\;\mu m \end{array}$$

These parameters were empirically determined and can be tuned for improved correction performance.

For CoMGrad, the Adam optimiser was employed with a learning rate of 0.01 and 30 iterations. At the start of each correction session, all Zernike coefficients were initialised to zero for both methods. For coordinate search, the estimated coefficient corrections were accumulated across successive correction rounds. In contrast, CoMGrad updated the coefficients iteratively using the Adam optimiser.

We evaluated the computation load for coordinate search and CoMGrad with the optimisation setups described above.

For coordinate search, the search complexity scales with the number of Zernike modes *N*, the number of search points per mode *S*, and the number of optimisation rounds *R*. Taking the computation load required to compute a single image quality metric from a given set of Zernike coefficients (the forward pass) as the unit computation load O , the total computation load $O_{\text{cs}}$ required for coordinate search can be approximated as:

(10) $$\begin{array}{l} O_{cs}\approx NSRO. \end{array}$$

Based on the analysis in Section 2.3, the computation load of CoMGrad is dominated by the forward and backward propagations involved in evaluating the coefficient–metric objective function. Consequently, the total computation load $O_{g}$ for CoMGrad can be approximated as:

(11) $$\begin{array}{l} O_{g}\approx 2N_{i}O \end{array}$$

where $N_{i}$ denotes the number of iterations used by the Adam optimiser. This assumes that the backpropagation process for computing the coefficient gradients has a similar computation load. Since the forward pass is dominated by Fast Fourier Transform (FFT) operations and the derivatives through the Fourier transform are approximately equivalent to an inverse FFT with element-wise multiplications, the backward pass has a comparable computational complexity. A comparison of the computation load between coordinate search and CoMGrad is obtained by comparing Eq. (10) and Eq. (11). The ratio of computation load between the two methods is therefore given by $NSR/2N_{i}$ . As a representative example, consider a common scenario where the number of effective Zernike modes is 12, covering up to the fourth radial order of Zernike modes, excluding piston, tip, and tilt. With 7 search points per mode and 9 optimisation rounds to account for coordinate search, and assuming 30 iterations for CoMGrad, the resulting ratio is approximately 12.6. Under these conditions, CoMGrad achieves more than an order-of-magnitude reduction of computation load compared to traditional coordinate search.

The above analysis of computation load does not fully reveal the actual computing time and speed difference, since the computation can be parallelised and accelerated on a GPU for both methods. For coordinate search, GPU acceleration was achieved by parallelising the calculation of the image quality metric within each search round. Specifically, N×S different combinations of Zernike coefficients were batched to compute the corresponding image metrics in parallel, where *N* is the number of modes, and *S* is the number of search points per mode. Since each round requires the intermediate corrected Zernike coefficients obtained from the previous round, different rounds cannot be parallelised. In contrast, GPU acceleration for CoMGrad was implemented by parallelising the correction of multiple small *en face* patches simultaneously. All experiments were conducted on a desktop computer with an Intel i5-13600 K CPU and an NVIDIA RTX 4080 Super GPU. GPU acceleration and parallelisation were primarily implemented using PyTorch.

In Section 3, further experimental results demonstrating CoMGrad’s speed improvement under the GPU-accelerated and parallelised implementation are presented.

## 3. Results

Experimental demonstration of the correction speed and performance of CoMGrad was conducted using two human-subject retinal imaging sessions. All experiments adhered to the principles of the Declaration of Helsinki. The first session evaluated small single-patch DWC for resolving human retinal photoreceptors. The subject’s right eye (OD) was dilated and imaged. The *en face* patch was located 1.5 mm nasal to the fovea. The second session demonstrated volumetric data DWC with sub-patches, evaluating the speed of CoMGrad for correcting a large amount of data with parallelisation. The subject’s left eye (OS) was dilated and imaged. The specific *en face* location being imaged on the retina is a 1.7 mm square centred at 1.5 mm nasal from the fovea.

### 3.1. Single-patch correction test on human retina photoreceptors

CoMGrad was first evaluated on a heavily aberrated *en face* patch of the human retinal photoreceptor layer. The correction was performed at the same *en face* location across 5 consecutively acquired volumes. Both CoMGrad and coordinate search used 42 Zernike modes, covering the second to eighth radial orders. Corrections were applied independently to each volume.

**Fig. 3.**
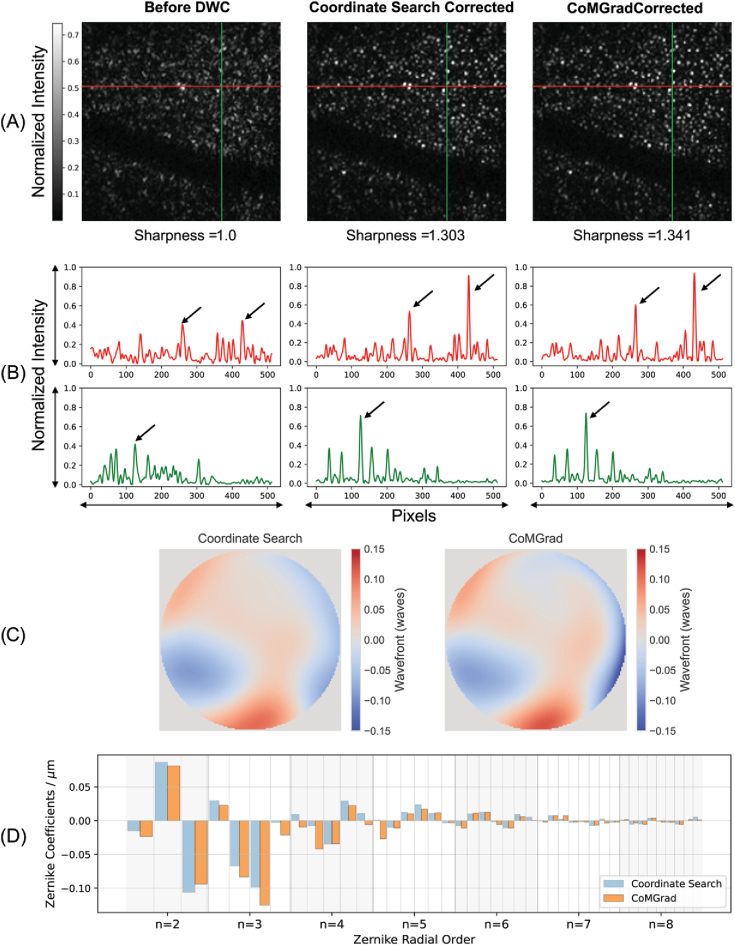
DWC applied to an *en face* photoreceptor layer. Panel (A): All images shown are registered and averaged from five volumes. Images have an original size of 128×128 pixels for DWC and are rescaled to 512×512 pixels for visualisation using cubic interpolation. Image metrics are calculated on the averaged and cubically rescaled images. Image pixel intensities are normalised to the range [0,1] by dividing all pixels by the maximum intensity among the three images. The intensity colour scale is clipped between the 1^st^ and 99.99^th^ percentiles of the pixel intensity distribution. This adjustment improves visual contrast because the high signal-to-noise ratio of the linear-scale OCT images results in a large dynamic range that would otherwise compress structural contrast. Coordinate search increases the image sharpness metric to 1.303, while CoMGrad achieves a higher optimal value of 1.341 for the image sharpness metric. Image sharpness metrics are normalised to the uncorrected image. Panel (B): The one-dimensional plots correspond to line profiles taken along the colored lines on the *en face* images. The vertical axes of the line profiles represent pixel intensities normalised to the maximum intensity of the corresponding image. Black arrows on the line plots highlight the increased image sharpness after DWC. Panel (C): Visualisation of the estimated aberrated wavefront after correction by coordinate search and CoMGrad. Panel (D): Zernike coefficient components of the estimated aberrated wavefront after correction by coordinate search and CoMGrad. The estimated wavefront and Zernike coefficients shown in Panels (C) and (D) are averaged over the corrections obtained from the five *en face* patches.

Figure 3 shows the *en face* photoreceptor images, corresponding image sharpness metrics before and after DWC using coordinate search and CoMGrad, and estimated wavefront distortion from both methods. The line profiles reveal a marked increase in peak-to-background ratio following correction, indicating that both methods effectively concentrate signal energy into narrower, higher-amplitude peaks. This behaviour is consistent with the observed improvement in the image quality metric. The estimated wavefront distortions obtained from both methods exhibit similar overall structures, with smoothly varying low-order aberrations dominating the wavefront shape. The corresponding Zernike coefficients show decreasing amplitudes with increasing radial order, which is consistent with the expected characteristics of ocular aberrations. Compared with coordinate search, CoMGrad produces slightly larger corrections in several higher-order modes, indicating a better capture of higher spatial frequency variations in the wavefront. The estimated Zernike coefficients from both methods are well within the correction limits described in Section 2.4. These limits were selected to cover the typical magnitude of ocular aberrations while also allowing sufficient margin to compensate for residual aberrations in the optical system. In practice, the measured coefficients remained significantly below the imposed bounds, indicating that the limits did not restrict the optimisation.

**Fig. 4.**
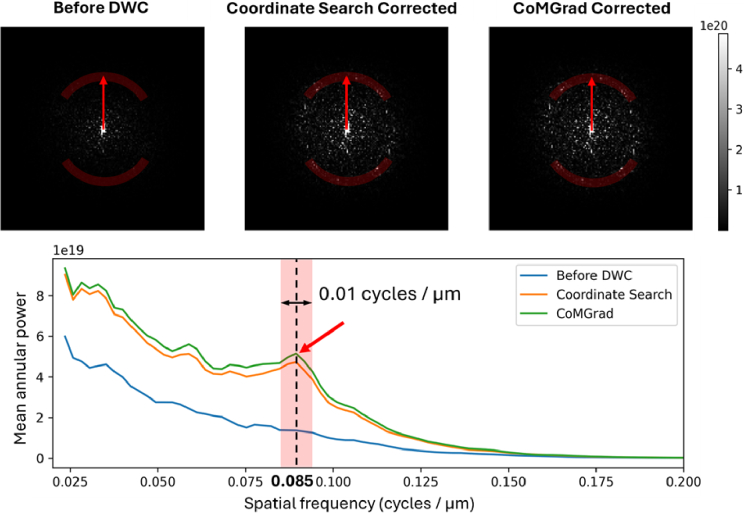
Visualisation and quantification of the Yellott’s ring before and after wavefront correction. Top panel: Power spectra of the *en face* patches in Fig. 3. The red arc indicates the region corresponding to Yellott’s ring spatial frequency. Only a partial annulus is shown for clarity, although Yellott’s ring extends over the full circular region. Increased prominence of the ring after correction reflects improved resolution of the photoreceptor spacing. Bottom panel: Mean annular power as a function of spatial frequency. The shaded region highlights the spatial frequency band corresponding to Yellott’s ring, centred at 0.085 cycles/µm with a bandwidth of 0.01 cycles/µm. Both correction methods increase the annular power within this band compared with the uncorrected case, indicating improved detection of the photoreceptor mosaic. CoMGrad’s correction produces the highest annular power in this region.

We additionally evaluated Yellott’s ring for DWC optimization as a clinically relevant indicator of photoreceptor mosaic resolution by capturing the characteristic spatial frequency corresponding to photoreceptor cone spacing [60,61]. Figure 4 exhibits the Yellott’s ring spectra and a 1-dimensional visualisation of mean annular power as a function of spatial frequency for the *en face* patches shown in Fig. 3.

**Fig. 5.**
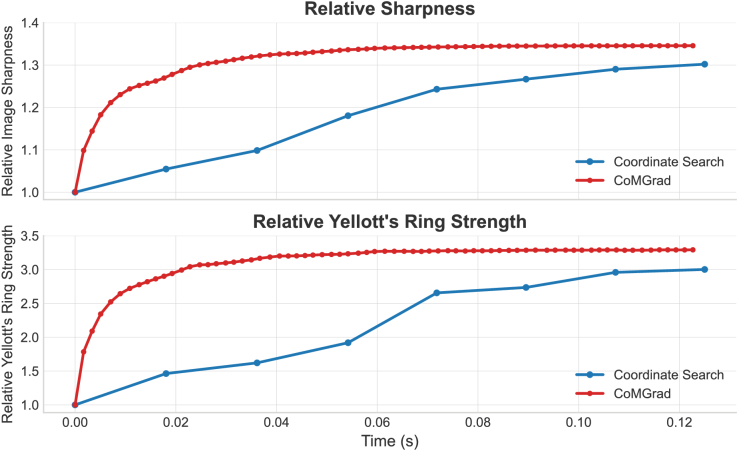
Temporal evolution of the averaged relative image sharpness and Yellott’s ring strength for coordinate search and CoMGrad when correcting a single *en face* patch of size 128×128 . The time stamps and corresponding relative image sharpness values are recorded and averaged across five volumes. At each time step, the sharpness metric represents the average sharpness of five *en face* patches, while Yellott’s ring strength is calculated from the averaged and interpolated image obtained from these patches. Both vertical axes are relative, relative to the starting point metric for each category. Both vertical axes show values normalised to the corresponding metric at the starting point of each method. Each blue marker represents one round of coordinate search, while each red marker represents the averaged image metric of each iteration of CoMGrad. The upper panel exhibits the temporal evolution of the image sharpness metric. For CoMGrad, the image sharpness rapidly converges and plateaus at approximately 1.35 after 30–40 iterations (≈0.05 s). In contrast, coordinate search exhibits a gradual increase in image sharpness with successive correction rounds, reaching a final value of approximately 1.31 after 9 rounds (≈0.14 s). The lower panel shows the temporal evolution of Yellott’s ring strength. Similar to the image sharpness trend, CoMGrad achieves faster convergence and a higher final Yellott’s ring strength compared with coordinate search.

In addition to visualisation of the corrected images, we recorded the execution time for each iteration of CoMGrad and for each round of coordinate search. We also recorded Yellott’s ring strength for intermediate correction results across iterations for both methods. Yellott’s ring strength was defined as the mean annular power within the region indicated by the red arcs in Fig. 4. Figure 5 shows the evolution of the relative image sharpness metric and Yellott’s ring strength as the correction progresses for both methods. Improvements in the sharpness metric are accompanied by increases in Yellott’s ring strength, indicating that the optimisation not only enhances the algorithmic image quality metric but also gradually improves clinically relevant image features.

### 3.2. Correction on volumetric data & visualisation

We further evaluated the speed advantage of CoMGrad on volumetric data.

Table 1 compares the correction quality and computational speed of the two methods under different numbers of target Zernike modes. All results are averaged over five independent correction runs for each method.

**Table 1.** Volumetric correction speed and quality of coordinate search & CoMGrad

Method	#Modes	Metric	Time / s
Coordinate Search	12	1.093	7.51
	25	1.101	14.45
	42	1.105	26.15
	63	1.106	42.41

CoMGrad	12	1.117	1.02
	25	1.135	1.05
	42	1.136	1.03
	63	1.148	1.07

The data used in Table 1 consist of seven sequentially acquired volumes, each containing seven adjacent *en face* slices of the target photoreceptor layer. Each volume had an *en face* size of 512×512 pixels and was divided into 16 patches of size 128×128 for digital wavefront correction (DWC). In total, DWC was applied to 16×7×7=784 *en face* patches. This experimental configuration mimics a mature STOC-T post-processing pipeline in which three-dimensional volumes are rapidly acquired, processed, layer-segmented, and subsequently corrected. Each image metric value shown in Table 1 was calculated from the full-size *en face* image reconstructed from the 16 corrected patches, after first averaging the corresponding patches across cross-sectional slices and volumes.

For coordinate search, 9 optimisation rounds with seven search points per mode were used. For CoMGrad, 30 optimisation iterations were performed for each correction. As the number of target Zernike modes increases, both methods exhibited an increase in the image quality metric, as expected, since higher-order aberrations can be corrected using additional modes. For coordinate search, the total correction time increased approximately linearly with the number of target Zernike modes. The approximately linear increase in runtime indicated that the GPU throughput was already saturated, and therefore, additional parallelisation did not provide further speed advantage. In contrast, the correction time for CoMGrad remained nearly constant as the number of modes increased, demonstrating that automatic differentiation efficiently computes gradients with respect to all coefficients using only two metric evaluations per iteration (forward and backward passes). For CoMGrad, a batch size of 49 images effectively utilised the available computational resources, where the throughput reaches a plateau.

Based on the results in Table 1, CoMGrad shows strong potential for correcting aberrations across multiple retinal layers and volumes within seconds. For a full volume containing 512 *en face* slices, CoMGrad is estimated to complete the correction in approximately 10 seconds under the current experimental conditions.

**Fig. 6.**
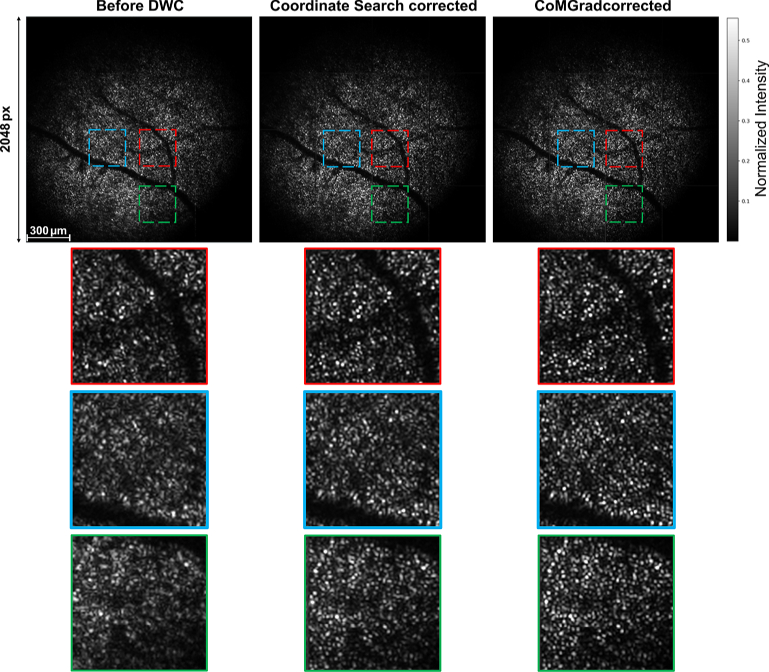
Visualisation of DWC results on the human retinal photoreceptor layer. The corrected volumetric data are identical to those used in Table 1. Top row: large-field *en face* images covering the STOC-T imaging field 1.7×1.7 mm. Each individual patch is averaged from 7 slices in depth and 7 volumes. Images are interpolated to 2048×2048 pixels using cubic interpolation from the original *en face* resolution of 512×512 for improved visualisation. The image intensity and the intensity scale bar employ the same normalisation and clipping scheme as Fig. 3. Red, blue, and green dashed squares mark three representative regions of interest (≈300 µm FOV) selected for detailed inspection. Bottom rows: corresponding zoomed-in views of the marked regions, illustrating local photoreceptor structure before and after correction. CoMGrad yields sharper photoreceptor mosaics and improved local contrast across the field compared to coordinate search, consistent with the quantitative improvements in image sharpness metrics.

Figure 6 provides a qualitative visualisation of DWC results on the photoreceptor layer of the human retina. The corrected volumes are identical to those used for the quantitative evaluation in Table 1. The number of Zernike modes used for correction is 63. The top row shows large-field *en face* images spanning the full imaging field of STOC-T of 1.7×1.7 mm. To improve visualisation, the displayed images are interpolated to a size of 2048×2048 pixels from the original *en face* resolution of 512×512 . Three representative regions of interest, each covering a FOV of approximately 300 µm, are indicated by red, blue, and green dashed squares. These regions sample different retinal locations across the imaging field and are selected to highlight different photoreceptor regions. The corresponding zoomed-in views are shown below each large FOV image, allowing a detailed comparison of photoreceptor visibility and local image quality.

Across all sampled regions, DWC leads to a clear enhancement in photoreceptor contrast and resolution. Compared to coordinate search, CoMGrad produces consistently sharper photoreceptor mosaics with higher local contrast and reduced background blur, particularly in regions distant from the centre. These visual improvements are consistent with the higher image sharpness metrics achieved by CoMGrad, shown in Table 1 for the case of 63 Zernike modes, and further demonstrate its effectiveness for wide-field, volumetric correction.

The superior correction quality of CoMGrad relative to coordinate search can be attributed to several factors. First, each isoplanatic patch within a large FOV *en face* slice experiences different aberrations. Coordinate search is more sensitive to optimisation hyperparameters, and variations in the initial aberrations across isoplanatic patches may require different parameter settings to achieve optimal performance. In practice, using a fixed set of parameters across all patches may lead to suboptimal correction in certain regions. Second, the performance of coordinate search depends on the number of correction rounds. While increasing the number of rounds can improve correction accuracy, it also leads to an increase in computational cost. In contrast, CoMGrad adapts naturally to spatially varying aberrations by exploiting global gradient information across all Zernike coefficients, enabling more reliable convergence without patch-specific parameter tuning or excessive iterations.

## 4. Discussions

We have presented CoMGrad, a coefficient–metric gradient-based method for digital wavefront correction in full-field swept-source OCT. CoMGrad exploits the differentiability of the mapping from Zernike coefficients to image quality metrics and combines reverse-mode automatic differentiation with the gradient-based optimiser Adam. Compared to zeroth-order optimisation methods, the first-order gradient-based optimisation scheme allows CoMGrad to perform high-resolution DWC on a large number of Zernike coefficients with low computation load. With GPU acceleration, CoMGrad enables aberration correction of volumetric data within seconds. Both theoretical analysis and experimental results demonstrate that CoMGrad achieves more than an order-of-magnitude speed improvement over the traditional coordinate search method.

Another advantage of CoMGrad is its stable convergence. In this work, the primary hyperparameters for CoMGrad are the learning rate and the number of iterations, both of which have only a minor influence on convergence within a reasonable range. In contrast, coordinate search requires the tuning of multiple parameters that can affect convergence, including the number of optimisation rounds, the optimisation limit for each Zernike mode, and the schedule by which these bounds are reduced across rounds. The sensitivity of coordinate search to these parameters likely originates from modal interactions on the image quality metric, whereby variations in certain Zernike modes influence the optimisation behaviour of others. Because coordinate search optimises each mode independently in a sequential manner, it cannot explicitly account for such modal interactions. Nevertheless, vulnerability to modal interactions is not inherent to zeroth-order optimisation methods such as SPGD, which can potentially overcome these interactions through the joint optimisation of coefficients. By contrast, CoMGrad leverages gradient information with respect to all Zernike coefficients simultaneously, enabling updates that follow the joint descent direction of the objective function in the full coefficient space. This global gradient information allows CoMGrad to converge more reliably in the presence of modal interactions.

While the current GPU-accelerated implementation of CoMGrad is based solely on the PyTorch framework, additional performance gains could potentially be achieved through native CUDA kernel optimisation. Direct CUDA programming offers finer control over memory management and thread scheduling, which may further accelerate CoMGrad and coordinate search processes. As a result, the execution time comparisons reported in this work may not fully reflect the peak performance attainable by either method. Nevertheless, although low-level hardware optimisations could slightly alter the absolute runtime and relative speed gap between the two approaches, both theoretical analysis and experimental results indicate that the primary advantage of CoMGrad lies in the analytical modelling from Zernike coefficients to image metrics, which enables the automatic differentiation framework and leads to local gradient calculation at low computation load. This property ensures superior scalability in high-accuracy digital wavefront correction scenarios that require a large number of Zernike modes.

We used coordinate search as a representative zeroth-order optimisation method for comparison. This algorithm is well established and relies only on metric evaluations rather than analytical gradients, making it a reliable baseline in sensor-less optimisation problems. The speed advantage of CoMGrad was therefore evaluated relative to this method. Other zeroth-order approaches, such as SPGD, may exhibit different performance characteristics in practice. However, since the analytical modelling between Zernike coefficients and the image metric is available in the FF-SS-OCT framework, CoMGrad can directly utilise the corresponding gradients through automatic differentiation, avoiding the need for measurement-based gradient approximation.CoMGrad establishes a gradient-based optimisation pipeline for digital wavefront correction. Further improvements may include exploring alternative image quality metrics to more effectively guide the optimisation process, as well as evaluating different optimisers to achieve smoother convergence near local optima and improved correction accuracy. Future work will also focus on further accelerating CoMGrad through direct CUDA kernel implementation. In addition, integrating AI-based automatic layer segmentation algorithms could reduce the number of *en face* slices required for digital wavefront correction, enabling near real-time DWC feedback for targeted retinal layers in full-field swept-source OCT systems.

## 5. Conclusions

This paper introduces CoMGrad, a coefficient–metric gradient-based digital wavefront correction method for full-field swept-source OCT. By exploiting the differentiable mapping between Zernike coefficients and image quality metrics in phase-stable full-field swept-source OCT data and leveraging reverse-mode automatic differentiation with the Adam optimiser, CoMGrad performs DWC efficiently using analytical local derivatives at low computation load. As a result, CoMGrad achieves more than a tenfold speed improvement over the traditional zeroth-order method coordinate search while maintaining high correction quality and stable convergence, making it well-suited for efficient volumetric digital wavefront correction.

## Data Availability

Data used for this publication is not available.
